# Supplementary material for: Health Literacy among Pregnant Women in a Lifestyle Intervention Trial
Source: Int J Environ Res Public Health. 2022 May 10;19(10):5808. doi: 10.3390/ijerph19105808 (PMC9141630; doi:10.3390/ijerph19105808)
Supplement: Supplementary file 1 [file ijerph-19-05808-s001.zip › ijerph-1685410-supplementary.pdf]

Table S1. Knowledge questionnaire.

**Topic**	**Question**
Gestational weight gain	Is it generally recommended for women of normal weight to gain 20 kilograms during pregnancy?
Portion size	Do pregnant women have to eat larger portions right from the start of their pregnancy to make sure that the baby gets enough food?
Alcohol	Can even small amounts of alcohol harm the unborn baby at any point during pregnancy?
Smoking	Does it harm the unborn child if people smoke around the pregnant woman (passive smoking)?
Physical activity	Does it harm the unborn child if women exercise during pregnancy?
Breast-feeding	Does breastfeeding work better the earlier a pregnant woman receives information about breastfeeding?
Water	Is tap water just as good for a pregnant woman as bottled mineral water?
Whole grains	Are wholegrain products usually the better choice if you want to eat pasta, bread or rice while pregnant?
